# Label-free electrochemical impedance biosensor to detect human interleukin-8 in serum with sub-pg/ml sensitivity

**DOI:** 10.1016/j.bios.2016.02.028

**Published:** 2016-06-15

**Authors:** R. Sharma, S.E. Deacon, D. Nowak, S.E. George, M.P. Szymonik, A.A.S. Tang, D.C. Tomlinson, A.G. Davies, M.J. McPherson, C. Wälti

**Affiliations:** a School of Electronic and Electrical Engineering, University of Leeds, Woodhouse Lane, Leeds LS2 9JT, UK; b School of Molecular and Cellular Biology, University of Leeds, Woodhouse Lane, Leeds LS2 9JT, UK; c BioScreening Technology Group, Astbury Building, University of Leeds, Leeds LS2 9JT, UK

**Keywords:** Label-free biosensor, Antibody mimetic protein, Interleukin-8, CXCL8, Electrochemical impedance spectroscopy, Point-of-care diagnostics

## Abstract

Biosensors with high sensitivity and short time-to-result that are capable of detecting biomarkers in body fluids such as serum are an important prerequisite for early diagnostics in modern healthcare provision. Here, we report the development of an electrochemical impedance-based sensor for the detection in serum of human interleukin-8 (IL-8), a pro-angiogenic chemokine implicated in a wide range of inflammatory diseases. The sensor employs a small and robust synthetic non-antibody capture protein based on a cystatin scaffold that displays high affinity for human IL-8 with a *K*_*D*_ of 35±10 nM and excellent ligand specificity. The change in the phase of the electrochemical impedance from the serum baseline, ∆*θ*(*ƒ*), measured at 0.1 Hz, was used as the measure for quantifying IL-8 concentration in the fluid. Optimal sensor signal was observed after 15 min incubation, and the sensor exhibited a linear response versus logarithm of IL-8 concentration from 900 fg/ml to 900 ng/ml. A detection limit of around 90 fg/ml, which is significantly lower than the basal clinical levels of 5–10 pg/ml, was observed. Our results are significant for the development of point-of-care and early diagnostics where high sensitivity and short time-to-results are essential.

## Highlights

• A label-free electrochemical impedance-based sensor for the detection of human interleukin-8 (IL-8) in full serum was developed.
• Detection limit of 90 fg/ml and time-to-result of 15 min was found.
• A large dynamic range of the sensor was observed, with sensor response linear *vs* logarithm of IL-8 concentration from 900 fg/ml to 900 ng/ml.
• The sensor employs a small and robust synthetic non-antibody capture protein, with high stability and excellent ligand specificity.
• Findings are particularly relevant for the development of point-of-care and early diagnosis sensors where high sensitivity and short time-to-results are essential.

## 1 Introduction

Modern healthcare systems rely heavily on *in vitro* diagnostics. The ability to detect biomarkers in body fluids at the point of care with high sensitivity and short time-to-result is becoming critical in a society placing increasing importance on both disease prevention, early diagnostics, and on stratified and individualised patient care. Interleukin-8 (IL-8; also referred to as CXCL8) is a chemokine that plays a pivotal role in acute inflammations and hence is an important biomarker for a range of diseases (Turner et al., 2014). During an injury or infection IL-8 is involved in the recruitment of neutrophils from blood vessels to the affected tissue (Hammond et al., 1995) promoting angiogenesis (Li et al., 2003). However, stimulants such as pro-inflammatory cytokines (*e.g*. TNF-α and interleukin-1), cellular stress, or bacterial and viral products, also trigger cells to express IL-8 proteins (Hoffmann et al., 2002), activating neutrophils (Zeilhofer and Schorr, 2000, Leung et al., 2001) that release their toxic intracellular contents causing damage to host tissue and resulting in acute inflammation states (Wright et al., 2010). Often, elevated IL-8 levels are also associated with the progression of numerous chronic diseases including rheumatoid arthritis, inflammatory bowel disease, psoriasis, palmoplantar pustulosis, idiopathic pulmonary fibrosis, acute respiratory distress syndrome (ARDS), atherosclerosis, central nervous system trauma, development of malignant cancer, and chronic liver disease (Mukaida, 2003, Skov et al., 2008, Waugh and Wilson, 2008, Apostolakis et al., 2009, Zimmermann et al., 2011). Therefore, the ability to monitor IL-8 concentrations with high accuracy in body fluids, *e.g*. serum, is an important prerequisite to enable early and accurate detection of severe illnesses, some of which result in progressive deterioration and consequent mortality.

Enzyme-linked immunosorbent assays (ELISAs) are currently the *de facto* standard for detecting IL-8 proteins in clinical diagnostics applications (Korostoff et al., 2011, Elsalhy et al., 2013, Necchi et al., 2014, Pedersen et al., 2015, Zhu et al., 2015). However, despite their widespread use, ELISA tests are limited in their applicability for point-of-care (POC) use as they generally require large and expensive instrumentation and additional reagents, are time-consuming with time-to-results of several hours, and are limited in sensitivity (Daniels and Pourmand, 2007). In healthy patients the IL-8 basal clinical level is 5–10 pg/ml (Benoy et al., 2004), and although most of the commercially available IL-8 ELISA kits state detection limits for human IL-8 of around 15±5 pg/ml, reproducible and reliable detection of small increases in concentrations in body fluids, a prerequisite for reliable early detection, remains a significant challenge. Alternative assays, such as the fluorescent-beads-based Luminex technology, are generally used to screen for enhanced levels of IL-8 rather than for quantitative measurements, mostly owing to reproducibility challenges (Djoba Siawaya et al., 2008, Gubala et al., 2012). In addition, label-free strategies for the detection of IL-8 were reported in the literature, including surface plasmon resonance (SPR)-based sensors that yielded a detection limit of 1.65 ng/ml IL-8 in human saliva (Yang et al., 2005). Recently, the detection of a few fg/ml of IL-8 in serum was demonstrated using a multi-step indirect sandwich immunoassay (Munge et al., 2011), where an electrode coated with a dense film of glutathione-protected gold nanoparticles modified with primary antibodies was used to capture human IL-8. For detection, super-paramagnetic beads coated with secondary antibodies and horseradish peroxidase were employed. However, time-to-result was a few hours, limiting their suitability for POC testing. Hence, to facilitate accurate, rapid and reliable testing at low cost to enable early diagnosis at POC, alternative approaches are required.

Electrochemical impedance spectroscopy (EIS) has been shown to provide a very promising biosensing approach with potentially very low limits of detection and time-to-results of minutes rather than hours. Examples offering limits of detection in the sub-pg/ml region include EIS-based sensors for the detection of soluble proteins such as IL-6, IL-2, and hCG (Berggren et al., 1998) and IFN-γ (Dijksma et al., 2001), albeit in buffer solutions. A direct label-free electrochemical biosensor for measuring IL-8 in clinical samples such as full serum at or below basal clinical levels, *i.e*. ≤5–10 pg/ml, has not been reported to date.

A typical EIS biosensor comprises a sensing element consisting of a conducting surface onto which capture molecules are immobilised which recognise and bind to the target protein of interest. The most widely employed capture molecules are antibodies. However, their interaction with solid surfaces can compromise their stability, which can lead to a loss of specificity and affinity (Butler et al., 1993, Conroy et al., 2009). Hence, small recombinant antibody fragments, nucleic acid aptamers, and constrained and unconstrained short peptides, inter alia, have been investigated as alternative capture molecules in biosensors (Evans et al., 2008, Johnson et al., 2008, Zeng et al., 2012; Yang et al., 2014; Zhou et al., 2014). Recently, we demonstrated the detection of anti-myc tag antibodies (Raina et al., 2015) by EIS employing capture molecules constructed from a small, robust and highly stable synthetic protein scaffold that displays two short peptide loops (Tiede et al., 2014). These short and highly constrained peptide loops were selected to provide the selective affinity to the target molecule. Here, we extend the use of these novel scaffold-based capture proteins to detect human IL-8 in full serum with sub-pg/ml sensitivity using a label-free EIS-based sensor.

## 2 Experimental section

### 2.1 Materials

Human IL-8 protein (ab73858) was purchased from Abcam, UK, diluted with deionised water (18.2 MΩ cm) to a stock concentration of 200 μg/ml and stored at −20 °C. Synthetic antibody mimetic proteins with high affinity to human IL-8 were selected *via* phage display. The capture protein coding region was sub-cloned into pET11 and recombinant protein was purified as previously described (Tiede et al., 2014, Raina et al., 2015). Monothiol-alkane-PEG-acid (HS-C_11_-(EG)_6_-OCH_2_-COOH) was purchased from Prochimia, Poland. Horse serum was sourced from Invitrogen, New Zealand, stored at 4 °C, and filtered with 0.22 μm filters supplied by Fisher Scientific, UK, prior to use. Sodium acetate was purchased from Fisher Scientific. 1-ethyl-3-(3-dimethylaminopropyl)carbodiimide hydrochloride (EDC), N-hydroxysuccinimide (NHS) and ethanolamine-HCl were purchased from GE Healthcare as a part of an amine coupling kit. All other reagents and solvents, unless stated otherwise, were purchased from Sigma Aldrich, UK. Deionised water (18.2 MΩ cm) from a Millipore water purification system was used to prepare all buffer solutions. Bare SPR disks with 48 nm of gold deposited on a layer of titanium, and compatible with the Autolab ESPRIT SPR system, were purchased from Metrohm Autolab, UK. Double junction Ag/AgCl reference ceramic wick electrodes were sourced from VWR, UK.

### 2.2 Methods

#### 2.2.1 Gold electrode cleaning

##### 2.2.1.1 SPR studies

SPR gold disks were sonicated for 10 min in a solution of 1% Triton X-100 in 100 mM NaOH, followed by 20 min sonication in 200 proof molecular biology grade ethanol.

##### 2.2.1.2 EIS studies

The gold electrodes used in the EIS studies, provided by Evatec AG (Switzerland), were fabricated by evaporating 20 nm of titanium and 80 nm gold onto a polished silicon/silicon oxide wafer. The evaporated electrodes (1 cm^2^) were sonicated twice in acetone for 10 min and subsequently rinsed in ethanol (200 proof).

#### 2.2.2 Functionalisation of gold electrode with monothiol-alkane-PEG acid SAM

The cleaned electrodes were rinsed with ethanol (200 proof) prior to immersion in 1 mM ethanolic solution of carboxylic-acid-terminated monothiol-alkane-PEG with 5% acetic acid. The electrodes were incubated for 48 h at room temperature to allow the assembly of a well-ordered self-assembled monolayer (SAM) on the gold electrode. Afterwards, the electrodes were rinsed in ethanol (200 proof) to remove excess molecules from the surface and dried under a stream of nitrogen.

#### 2.2.3 Immobilisation of the synthetic binding proteins on the SAM layer

Electrodes functionalised with SAMs were mounted either in an electrochemical cell for EIS studies or on a hemi-cylinder for SPR measurements. To attach the synthetic binding proteins on the SAM surface, the exposed area of the gold electrodes intended for EIS measurements was rinsed with 100 mM 2-(N-morpholino)ethanesulfonic acid (MES) buffer at pH 5.5, while for SPR-based studies, the buffer was injected into the channel until a stable SPR baseline was established. The carboxylic acid group of the SAM layer was activated by exposing the electrode surface for 15 min to a 1:1 mixture of 400 mM EDC and 100 mM NHS in 100 mM MES buffer at pH 5.5.

The activated surface was then washed with 100 mM MES buffer pH 5.5 to remove excess EDC/NHS from the surface followed by rinsing with 10 mM acetate buffer at pH 5.5. Subsequently, the surface was incubated for 30 min in the same buffer containing the capture proteins at a concentration of 10 μg/ml. Finally, the surface was exposed to a 1 M ethanolamine solution at pH 8.5 for 15 min to quench any residual activated sites on the SAM layer.

#### 2.2.4 Surface plasmon resonance

SPR experiments were performed in a two-channel Autolab ESPRIT system (Autolab, Netherlands). The temperature of the system was kept constant at 22 °C using a recirculating water bath (R-150 Grant) connected to the SPR system.

##### 2.2.4.1 Capture protein attachment

Optimal pH conditions for the immobilisation of the synthetic non-antibody binding proteins were established in 10 mM buffer solutions with a range of pHs from 8 to 4.5. Phosphate buffer was used for pH 8.5–6, while for pH 5.5–4.5, acetate buffer was employed. The gold electrode functionalised with the SAM and mounted in a SPR flow channel was subjected to a buffer solution, starting with the highest pH *i.e*. 8.5. Binding proteins at 10 μg/ml in the same buffer were then allowed to adsorb onto the surface for 400 s, while monitoring the SPR signal. After each pH, the surface was regenerated with 10 mM NaOH solution to remove the adsorbed binding proteins completely from the SAM. A new baseline was then established with the buffer solution at another pH and the experiment repeated.

##### 2.2.4.2 IL-8 detection

The gold electrodes with immobilised binding proteins and blocked with ethanolamine were washed with 100 mM phosphate buffer at pH 7 and a stable baseline was established. IL-8 proteins diluted in 100 mM phosphate buffer pH 7 to a concentration of 1 μg/ml was applied to one of the channels, while the second channel was used as a reference. The SPR signal was monitored to detect binding of the IL-8 proteins to the surface.

#### 2.2.5 Electrochemical impedance spectroscopy (EIS)

All EIS experiments were carried out in a three-electrode electrochemical cell, comprising a double junction Ag/AgCl as reference electrode, a platinum wire as a counter electrode and a cleaned gold surface as the working electrode. The working electrode was 2 mm in diameter and its distance from the reference electrode during EIS measurements was about 5 mm. The reference electrode was stored in a 3.5 M solution of KCl when not in use. The electrochemical cell was placed in a Faraday cage to minimise interference from any external electrical noise.

The gold electrodes were mounted in an electrochemical cell. The electrodes were then washed twice in the electrolyte (100 mM phosphate buffer pH 7 or serum, as appropriate) before 800 μl and 500 μl of fresh buffer and serum, respectively, was added. Electrochemical impedance measurements were performed using a BioLogic VSP potentiostat and EC-lab software provided with the system was used to record and analyse the response data. All EIS measurements were performed by applying a 30 mV sinusoidal ac potential to the working electrode at frequencies from 100 kHz–50 mHz (7 points/decade), unless stated otherwise, superimposed on a dc potential between 0 mV and 80 mV, and 0 mV and 100 mV, for measurements in buffer and serum, respectively. Before proceeding with the biosensing EIS measurements, the SAM-functionalised electrodes were subjected to an EIS measurement at a 200 mV dc potential for ten cycles in order to transform a well packed SAM into a leaky capacitor (Boubour and Lennox, 2000) and to reach a stable EIS baseline. All potentials are reported *vs* Ag/AgCl. EIS scans were repeated for five cycles.

#### 2.2.6 Differential scanning calorimetry

The thermal stability of the non-antibody binding proteins as well as the empty scaffold protein was assessed using a VP-DSC MicroCalorimeter (GE Healthcare). Each sample was dialysed against 1× phosphate buffered saline (PBS) pH 7.4 and diluted to a concentration of 0.5 mg/ml. After degassing, the heat capacity of the samples was measured from 10 °C to 120 °C at a scan rate of 90  °C/h.

#### 2.2.7 Bio-layer interferometry (BLI)

The binding kinetics between the non-antibody binding proteins and human IL-8 was measured using a bio-layer interferometer (BLItz, Pall Fortebio). The measurements were carried out using the Ni-NTA dip and read biosensors. A baseline was first established in 1× PBS buffer by measuring the response of the sensor for 30 s. Binding proteins at 100 µg/ml concentration in 1× PBS were then immobilised on the sensor *via* their C-terminus eight-histidine tags for 2 min. To obtain the association phase, the sensor was rinsed with 1× PBS for 30 s, before being exposed to different concentrations of IL-8 proteins in 1× PBS buffer for 5 min. Subsequently, 1× PBS buffer was applied and the dissociation phase was recorded. The data analysis was carried out using ProFit software (Quansoft, Switzerland).

#### 2.2.8 Mass spectrometry

Non-antibody binding proteins in 1× PBS were buffer exchanged into 50 mM ammonium acetate using Zeba spin columns (7k MWCO; Thermo Scientific) and samples of 20 µl containing 20 µM proteins were analysed on a Synapt HDMS (Waters UK Ltd.) electrospray mass spectrometer.

## 3 Results and discussion

### 3.1 IL-8 specific capture molecules

We have previously developed non-antibody-based binding molecules employing small scaffold proteins (*M_w_*∼12 kDa) that display two short, constrained peptide loops, which provide high and specific affinity for the target protein. A detailed discussion of these antibody mimetics is given elsewhere (Tiede et al., 2014, Raina et al., 2015). In brief, a highly diverse antibody mimetic library where both binding loops were randomised was generated (diversity >10^10^), and phage display was employed to select binders with affinity for human IL-8. Four clones with high affinity for human IL-8 proteins were identified and initially assessed *via* a phage ELISA assay (Fig. S1). The binding protein showing the strongest affinity to IL-8 was chosen as the capture molecule for the EIS biosensor.

The IL-8-specific binding protein was characterised in detail, and its molecular mass determined using mass spectroscopy was found to be 13.52 kDa, agreeing with the expected value (Fig. S2). A high stability of the capture molecule is of importance for biosensors to ensure sensor stability and device shelf-life, and so differential scanning calorimetry (DSC) measurements were carried out to assess stability. The heat capacity *C*_*p*_ is shown in Fig. S3 as a function of temperature between 20 °C and 110 °C. A distinct melting peak at *T*_*m*_=82 °C was observed for the IL-8 binding proteins, demonstrating the very high thermo-stability. For comparison, the heat capacity of the empty scaffold (*T*_*m*_=101 °C) is shown in the same figure. The reduced melting temperature of the binding protein compared to the empty scaffold is likely to be due to the changes in the structure of the scaffold protein where the binding loops were introduced. Similar observations were reported for binding proteins designed to capture anti-myc tag antibodies (Raina et al., 2015).

The limit of detection that can be achieved by a label-free biosensor depends on the affinity between the capture molecule and the target protein, and hence the performance of the IL-8 binding molecule was investigated using bio-layer interferometry (BLI). The binding proteins were attached to a pre-activated Ni-NTA BLI sensor tip at a concentration of 10 µg/ml *via* the eight-histidine tag of the protein located at the C-terminal. The sensor was then exposed to different concentrations of human IL-8 proteins in PBS pH 7.0 for 5 min each to ensure saturation was reached, while the association sensogram was recorded. After each association, the sensor was exposed to PBS and the dissociation phase was recorded.

**Fig. 1:**
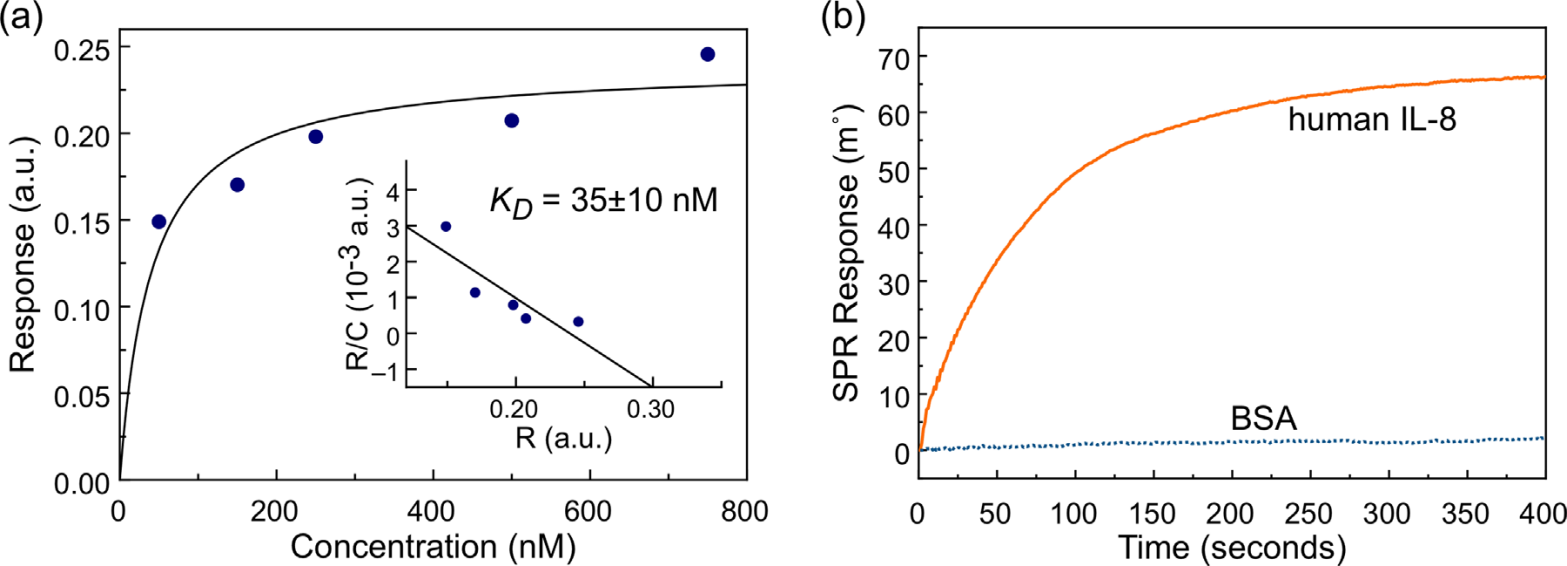
(a) Binding response at equilibrium of human IL-8 binding to non-antibody capture molecule immobilised onto the sensor surface detected using bio-layer interferometry. The solid line shows the least-square fit of the Langmuir binding isotherm to the linearised data. Inset: linearised form of binding data where the *y*-axis *R*/*C* corresponds to the sensor response at equilibrium (*R*) divided by human IL-8 concentration (*C*), and the *x*-axis to the sensor response at equilibrium (*R*). (b) Sensogram showing change in the SPR angle of the sensor functionalised with the binding protein to both human IL-8 and BSA from the 100 mM phosphate buffer pH 7.4 baseline.

Fig. 1a shows the response of the sensor at equilibrium as a function of IL-8 concentration. The solid line shows a least-square fit of the Langmuir binding isotherm *R*_*eq*_=*CB_max_*/(*C*+*K_D_*) to the response of the sensor at equilibrium, *R*_*eq*_, in linearised form (Scatchard plot, shown in the inset), where *C* is the concentration of the human IL-8, *B_max_* the sensor response at saturation, and *K*_*D*_ the dissociation constant. The fit revealed a *K*_*D*_ of 35±10 nM, demonstrating the high affinity of the binding protein to human IL-8. This compares well with the affinities of two native human IL-8 receptor proteins CXCR1 and CXCR2 with reported solution-phase *K*_*D*_ of 5 nM and 2.5 nM, respectively (Nasser et al., 2007), in particular when taking into account that the IL-8 binding proteins reported here were attached to a solid surface which generally leads to an order of magnitude increase of *K*_*D*_ compared to solution-phase *K*_*D*_.

### 3.2 Sensing element

**Fig. 2:**
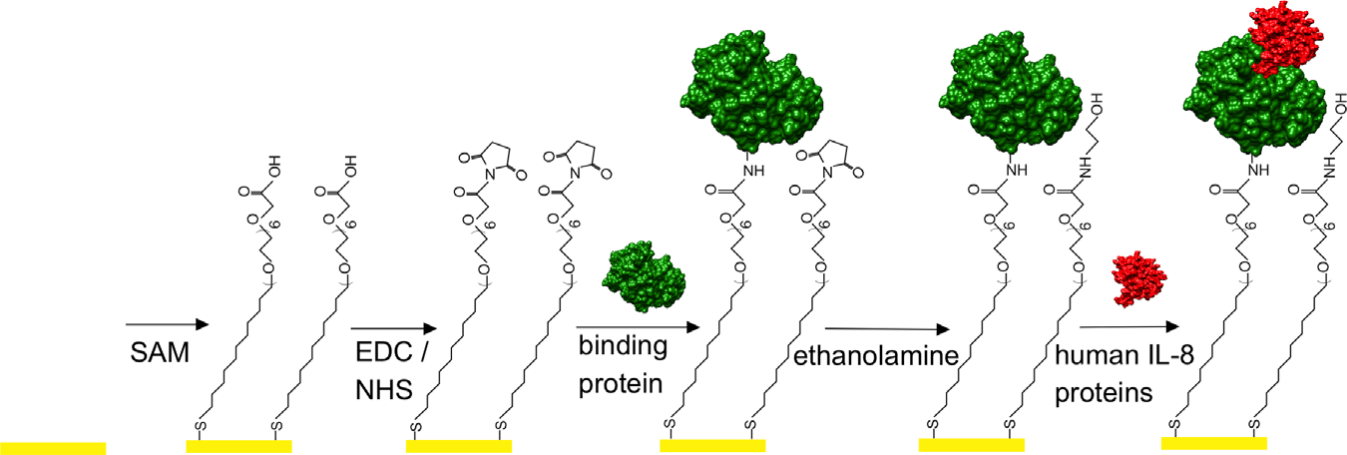
Schematic illustration of the biosensor assembly. A monothiol-alkane-PEG acid SAM is first assembled on a gold electrode. The carboxylic acid groups of the SAM are then activated with EDC/NHS to which the non-antibody capture proteins are covalently attached. Following deactivation of residual activated acid sites with ethanolamine, the sensor surface is challenged with fluids containing human IL-8 protein and the electrochemical impedance of the sensor is monitored.

The different components of the sensing element of the biosensor developed in this work are shown in Fig. 2, and comprise a gold electrode coated with a SAM of monothiol-alkane-PEG-acid (HS-C_11_-(EG)_6_-OCH_2_-COOH). The PEG moieties of the SAM yield a highly anti-fouling surface which prevents any non-specific binding of unwanted molecules to the sensor element from the sample fluid (Herrwerth et al., 2003). The non-antibody capture protein specific to IL-8 is covalently attached to the SAM *via* carboxylic acid groups pre-activated with EDC and NHS.

To establish optimal coupling conditions for immobilising the capture proteins onto the SAM surface, a range of SPR experiments were carried out at different pH. Binding proteins at a concentration of 10 μg/ml in sodium acetate and phosphate buffers of pH between 4.5 and 8 were applied to the SAM-functionalised gold electrodes, and the change in the SPR angle was monitored (Fig. S4). Only a very small change in SPR angle (∼60 m°) was observed when the binding proteins were in buffers at pH≥7.5. Upon lowering the pH, the response gradually increases and then starts to saturate at pH≤5.5 where changes in the SPR angle of about 430 m° were measured. We note that the onset of the saturation coincides with the pH at which the overall charge of the IL-8 capture molecule becomes positive.

For all following sensor experiments, the activated carboxylic acid SAM-functionalised gold electrodes were exposed to 10 µg/ml of IL-8 capture protein in 10 mM sodium acetate buffer, pH 5.5, and the residual activated carboxylic acids were capped with ethanolamine. To test the specificity of the sensor element, fully functionalised surfaces were exposed to human IL-8 proteins at 1 μg/ml in 100 mM phosphate at pH 7 and the change in the SPR angle was monitored in real-time. A 66 m° shift from the baseline (100 mM phosphate buffer pH 7) was recorded at saturation. This contrasts with the response of less than 2 m° that was observed when the sensor was challenged with 5 µg/ml bovine serum albumin (BSA), clearly demonstrating the specificity of the binding protein for human IL-8 (Fig. 1b). The full SPR sensogram, including the capture molecule immobilisation on the SAM functionalised electrode and subsequent detection of human IL-8, is shown in Fig. S5a.

### 3.3 Label-free detection of IL-8 using electrochemical impedance spectroscopy

EIS is a powerful tool for the label-free detection of target proteins. For biosensors employing molecular layers at the interface between the sensor electrode and the sample fluid, the electrochemical impedance is governed by the impedance of the sensor–electrolyte interface, which is generally dominated by capacitive contributions. The interface impedance is the sum of the contributions from the SAM, the capture protein layer, the internal Stern layer, and the diffused Gouy and Chapman layer that extends into the electrolyte solution (Rampi et al., 1998, Bard and Faulkner, 2000). Changes to any of these components, such as binding of the biomarkers to the capture molecules, results in an overall change in impedance, which is then reflected in the change of the amplitude *Z* and the phase *θ* of the sensors' electrochemical impedance. For sensors where a highly packed SAM with low leakage and a high dielectric constant is employed at the interface, the binding of the ligand to the capture molecules predominately changes the capacitance associated with the Stern layer, which forms the basis of capacitive electrochemical sensors (Berggren et al., 2001). This change in capacitance is generally attributed to the displacement of water molecules and solvated ions further away from the sensor surface (Berggren et al., 1998). In contrast, for sensors employing less densely packed SAMs, defects such as pinholes in the SAM can contribute significantly to the overall impedance, and in fact any change in the density or size of the defects can lead to substantial changes in the electrochemical impedance (Zaccari et al., 2014). Upon binding of target proteins to the capture molecules attached to the SAM, the local environment of the SAM may change, potentially leading to changes in SAM defects. This in turn leads to a change in the electrochemical impedance, which can be measured.

The phase of the impedance, *θ*(*ƒ*), has previously been shown to be a reliable and sensitive measure to monitor the binding of target proteins to the sensor surface as well as changes in the sensor interface (Evans et al., 2008, Raina et al., 2015). Hence, changes in the phase of the impedance, Δ*θ*(*ƒ*), were employed here for the detection of human IL-8 proteins, but also for evaluating the assembly of monothiol-alkane-PEG SAMs on the gold electrode, and subsequent immobilisation of the capture proteins during sensor fabrication.

#### 3.3.1 SAM assembly

**Fig. 3:**
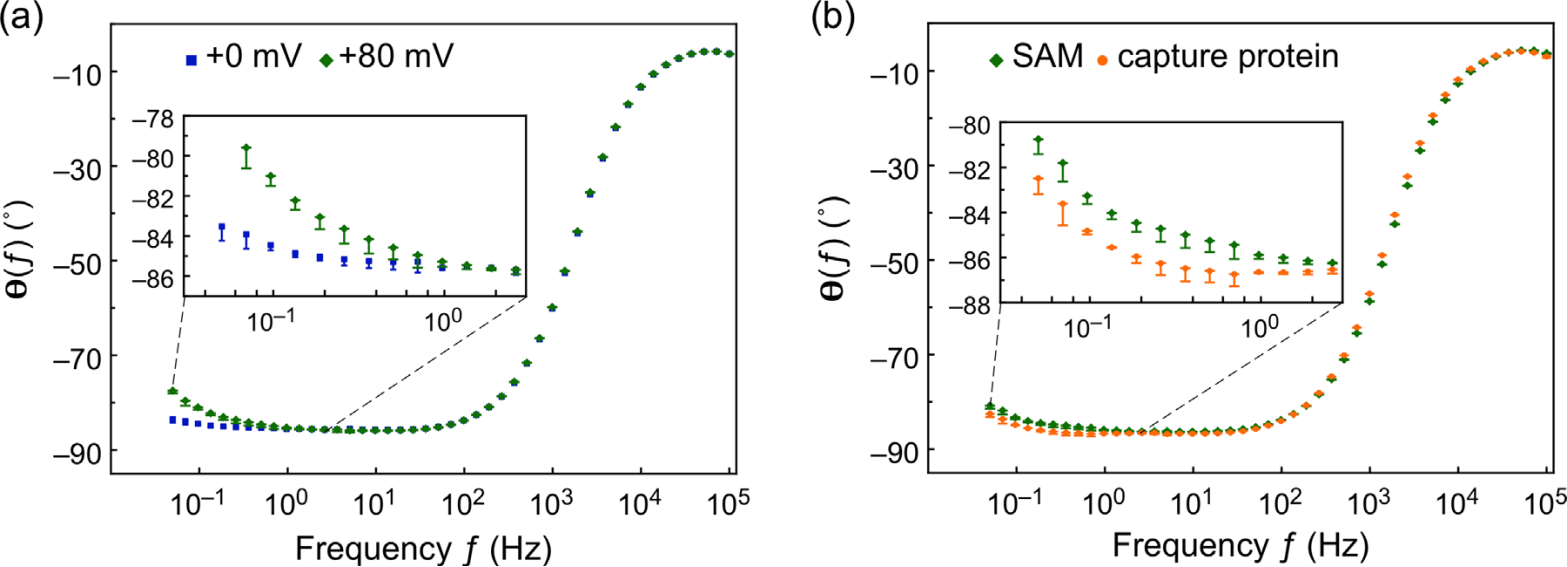
EIS Bode plots showing (a) the phase *θ*(*ƒ*) after the formation of the monothiol-alkane-PEG acid SAM on the gold electrode at 0 mV and +80 mV dc potential, and (b) the phase *θ*(*ƒ*) after immobilisation of the non-antibody capture molecules on the SAM in comparison to the SAM only at a dc offset potential of +80 mV *vs* Ag/AgCl. The EIS measurements were conducted in 100 mM phosphate buffer at pH 7 and the data shown represent the average *θ*(*ƒ*) of five EIS scans. Corresponding Nyquist plots are shown in Fig. S7.

The monolayer quality was assessed by EIS and spectra were taken between 100 kHz and 50 mHz at Ag/AgCl potential. The average phase of the impedance calculated from five different scans is shown in Fig. 3a. A dominating capacitive behaviour of the SAM was observed at low frequencies where *θ*(*ƒ*) is approaching −90°, indicative of a densely packed SAM formed on the electrode that very effectively blocks the current. On average, the phase of the impedance at 0.1 Hz, *θ*(*ƒ*)_0.1 Hz_, was −84±2°.

Non-zero dc potentials ≥+150 mV were reported to induce pinholes into a SAM and thus to transform a well-packed SAM into a leaky capacitor with correspondingly increased *θ*(*ƒ*) (Boubour and Lennox, 2000). Comparable results were obtained in recent work where an increased *θ*(*ƒ*) was found at low frequencies at higher dc potential, and the EIS sensors were found to be more sensitive with increased *θ*(*ƒ*) (Raina et al., 2015). A similar increase in the low-frequency phase of the electrochemical impedance was observed here when the dc potential of SAM functionalised electrodes was raised from +0 mV to +80 mV. This is shown in Fig. 3a, where the average of *θ*(*ƒ*)_0.1 Hz_ taken across five devices increased to −81±2° at +80 mV from −84±2° recorded at 0 mV dc bias.

#### 3.3.2 Capture protein immobilisation

Following the SAM assembly, capture proteins were immobilised on the activated carboxylic acid SAM surface, followed by quenching of residual sites with ethanolamine. Similar to above, the phase of the electrochemical impedance was measured at 0 mV and +80 mV dc potential from 100 kHz to 50 mHz. The phase of the sensor was found to decrease towards −90° at frequencies below 1 Hz, with a difference of almost 2° at 0.1 Hz. In contrast, the spectrum above 1 Hz was not affected. The results are shown in Fig. 3b (80 mV dc bias) and Fig. S6 (0 mV dc bias), where, after the attachment of the capture proteins, *θ*(*ƒ*)_0.1 Hz_ decreased from −83.3° to −84.8°, and from −85.5° to −86.6°, at 80 mV and 0 mV dc bias, respectively. This suggests that the capture proteins and ethanolamine form a densely populated layer, resulting in an increase in the capacitive behaviour of the sensor.

#### 3.3.3 IL-8 detection

**Fig. 4:**
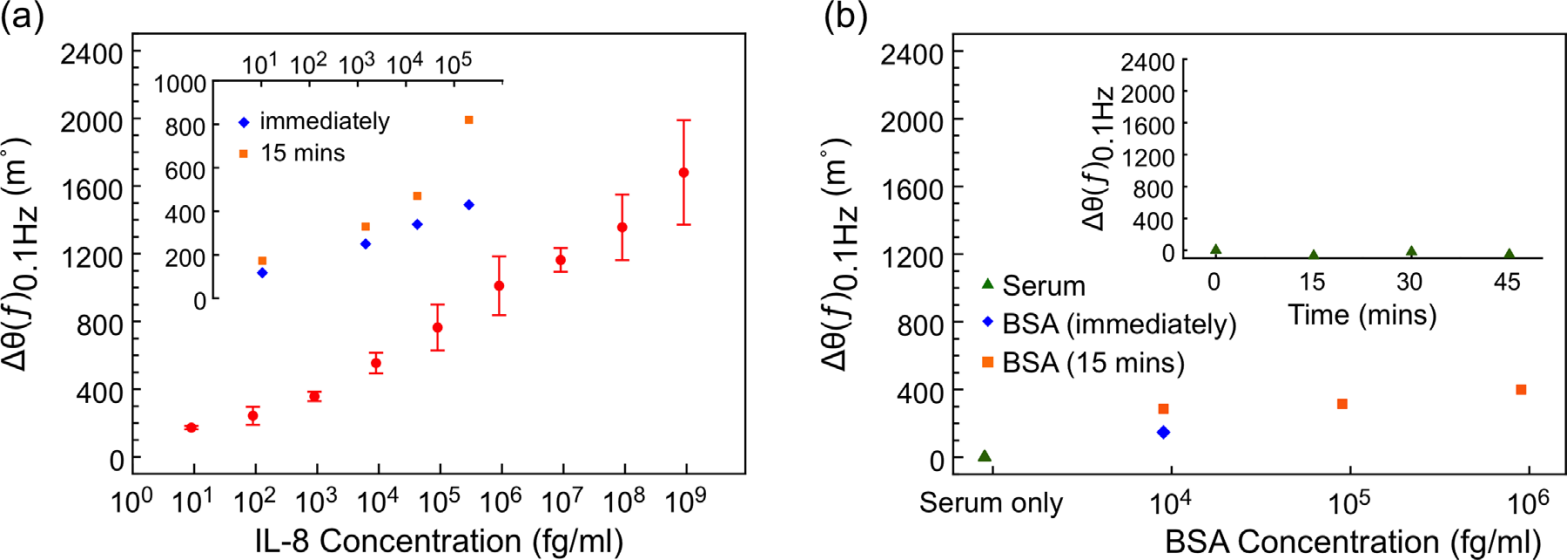
(a) EIS sensogram showing the change in phase from the baseline at 0.1 Hz, ∆*θ*(*ƒ*)_0.1 Hz_, of the sensor response *vs* IL**-**8 concentration between 9 fg/ml and 900 ng/ml (a representative Nyquist plot for one of the devices is shown in Fig. S8). Inset: effect of IL-8 incubation time on ∆*θ*(*ƒ*)_0.1 Hz_ from the baseline, with measurements taken immediately and after 15 min of incubation. (b) EIS sensogram showing the response of the sensor when exposed to IL-8 free serum and BSA-spiked serum. The inset shows the variance of the sensor signal over time. All EIS scans were performed at a dc offset of +100 mV *vs* Ag/AgCl.

Before exposing the sensor devices to different concentrations of human IL-8 protein spiked into horse serum, the sensors were incubated in blank serum for 15 min to establish a baseline. 50 μl of the serum in the electrochemical cell was then replaced with serum containing human IL-8 to achieve the desired concentration. The phase of the electrochemical impedance *θ*(*ƒ*)_0.1 Hz_ was immediately measured at a +100 mV dc offset potential and then after 15 min of IL-8 incubation, and the difference in phase with respect to the baseline, Δ*θ*(*ƒ*)_0.1 Hz_, was determined. Multiple devices were fabricated and exposed to different starting concentrations of IL-8; the Δ*θ*(*ƒ*)_0.1 Hz_ response of a typical sensor device is shown in Fig. 4a.

The performance of the sensor was investigated across a concentration range between 9 fg/ml and 900 ng/ml. A small change in *θ*(*ƒ*)_0.1 Hz_ of 173 m° from the serum baseline was observed at an IL-8 concentration of 9 fg/ml (1 fM), and when the concentration was increased by 10-fold, a small further increase in *θ*(*ƒ*)_0.1 Hz_ of 70.6 m° was recorded. Above this IL-8 concentration, the sensor recorded signal was comparatively high, and a linear response versus logarithm of IL-8 concentration across the entire concentration range was observed with a slope of 220.4 m°/decade.

For biomarker detection in complex clinical samples such as serum it is important for the sensor devices to show no or minimal response to any molecules other than the desired target proteins. To test the non-specific response, the devices were exposed to horse serum that was not spiked with human IL-8 protein, and the phase of the electrochemical impedance *θ*(*ƒ*)_0.1 Hz_ was measured at a +100 mV dc offset potential immediately, and 15 min after the serum injection. 50 μl of the total serum volume was then replaced with fresh serum and the sensor device incubated for a further 15 min before measuring the change in phase, Δ*θ*(*ƒ*)_0.1 Hz_. This was repeated for an additional three cycles and the average Δ*θ*(*ƒ*)_0.1 Hz_ taken from two independent devices is shown in the inset of Fig. 4b. We note that the measured phase fluctuated by less than 60 m° over the course of 45 min, demonstrating that the device is highly stable across the time-scale relevant for the EIS sensors discussed here.

The non-specific binding of the sensor was further examined by subjecting it to serum samples spiked with BSA at different concentrations. A change in *θ*(*ƒ*)_0.1 Hz_ of 286 m° was observed when the device was exposed to 9 pg/ml of BSA, which increased further by 30 m° upon further incubation for 15 min. For a higher concentration of BSA, *i.e*. 900 pg/ml, a change in *θ*(*ƒ*)_0.1 Hz_ of 400 m° was observed after 15 min incubation (Fig. 4b), which is significantly lower than the signal measured for the same concentration of human IL-8. A similarly small change in the *θ*(*ƒ*)_0.1 Hz_ was observed when the sensor was exposed to human interleukin-6 (IL-6) at concentrations between 10 fg/ml and 100 ng/ml (*θ*(*ƒ*)_0.1 Hz_<350 m° at 100 ng/ml; Fig. S9), demonstrating the specificity of the sensor.

## 4 Conclusions

We have demonstrated a label-free biosensor based on EIS to detect human IL-8 protein spiked into full serum at clinically relevant concentrations with very high sensitivity and a time-to-result of 15 min. The sensor employs a small and robust antibody mimetic as the capture molecule, which was selected from a highly diverse library using phage display. The capture molecule was found to be highly selective for human IL-8 with a *K*_*D*_ of 35±10 nM, and was found to be very stable with a melting temperature of 82 °C.

The change in the phase of the electrochemical impedance at 0.1 Hz, ∆*θ*(*ƒ*)_0.1 Hz_, was used as the measure to quantify the binding of IL-8 proteins to the surface-immobilised non-antibody capture molecules, and a detection limit of around 90 fg/ml of IL-8 in full serum was demonstrated. Furthermore, a linear relationship between ∆*θ*(*ƒ*)_0.1 Hz_ and the logarithm of the IL-8 concentration in serum was observed between 900 fg/ml and 900 ng/ml, *i.e*. over six orders of magnitude.

These findings are of particular significance for POC diagnostics, where high sensitivities and detection limits well below basal clinical levels, as well as short time-to-results, are required to enable early diagnostics.

## Data statement

Data supporting this study are provided in the results section of this paper and as Supplementary information accompanying this paper.
