# Supplementary material for: Label-free electrochemical impedance biosensor to detect human interleukin-8 in serum with sub-pg/ml sensitivity
Source: Biosens Bioelectron. 2016 Jun 15;80:607–13. doi: 10.1016/j.bios.2016.02.028 (PMC4785862; doi:10.1016/j.bios.2016.02.028)
Supplement: Supplementary file 1 — Supplementary material [file mmc1.docx]

**Supplementary Information**


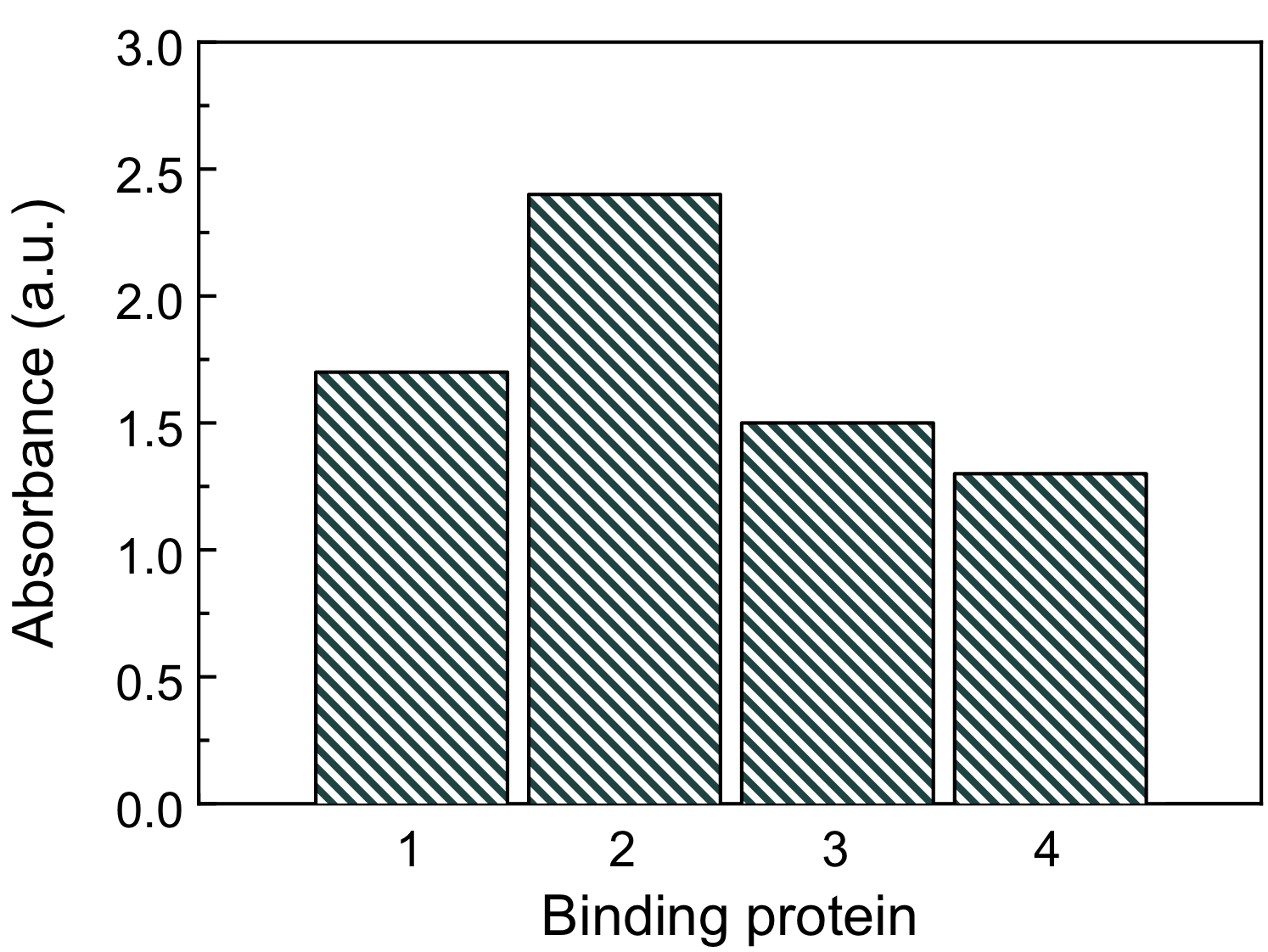


**Fig. S1** Absorbance measured at 650 nm showing the normalised ELISA response of the phage particles displaying the four selected binding proteins when exposed to 200 nM of human IL-8 proteins. Binding protein 2 was chosen as the capture protein for the biosensor.


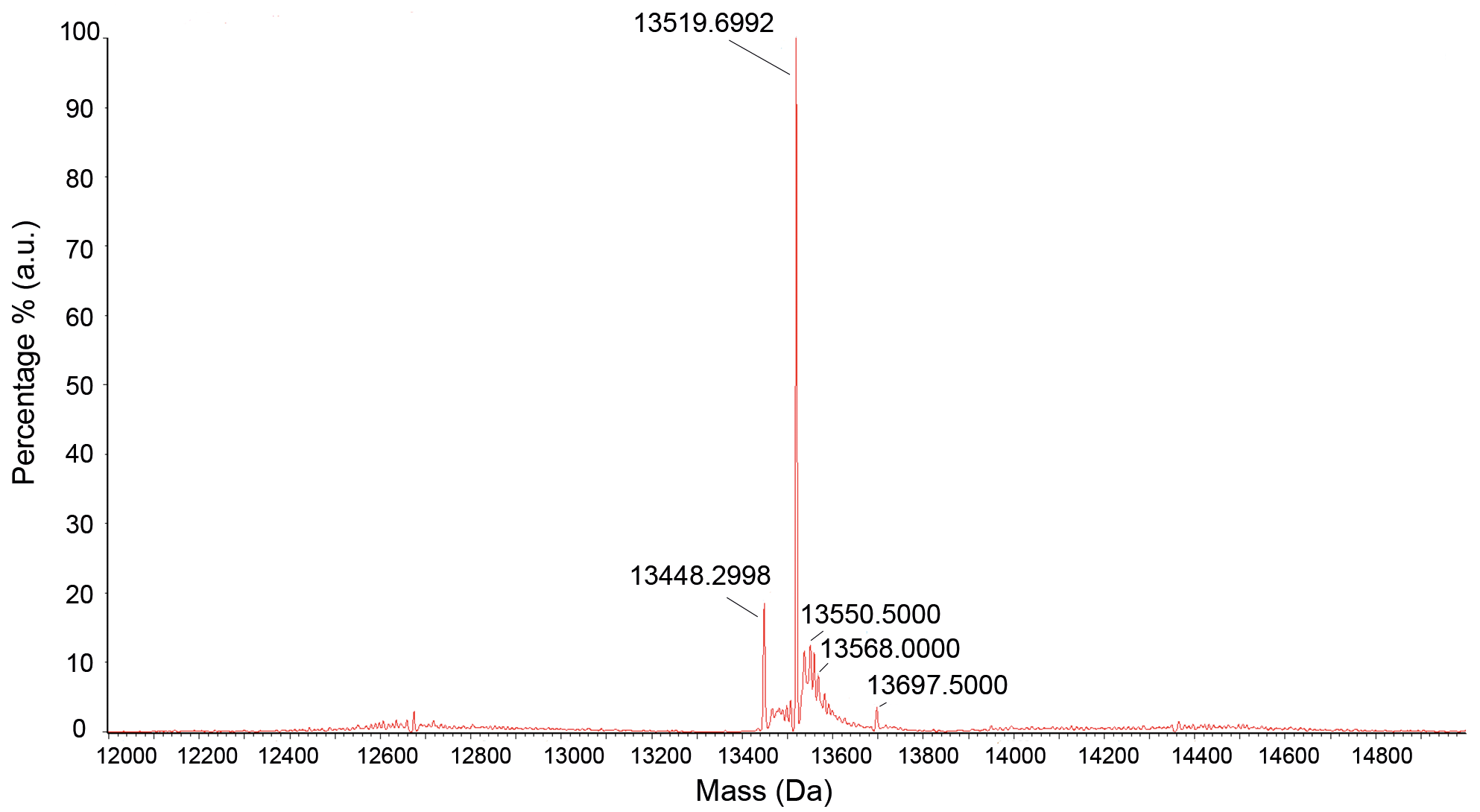


**Fig. S2** Mass spectrum of the non-antibody capture protein. The molecular weight of the IL-8 specific binding protein was found to be 13.520 kDa, matching the expected value. The predominant species was confirmed to be lacking alanine with a minor component lacking N-terminal Met. Additional peaks in the spectrum are the results of common modifications associated with cytoplasmically expressed proteins.


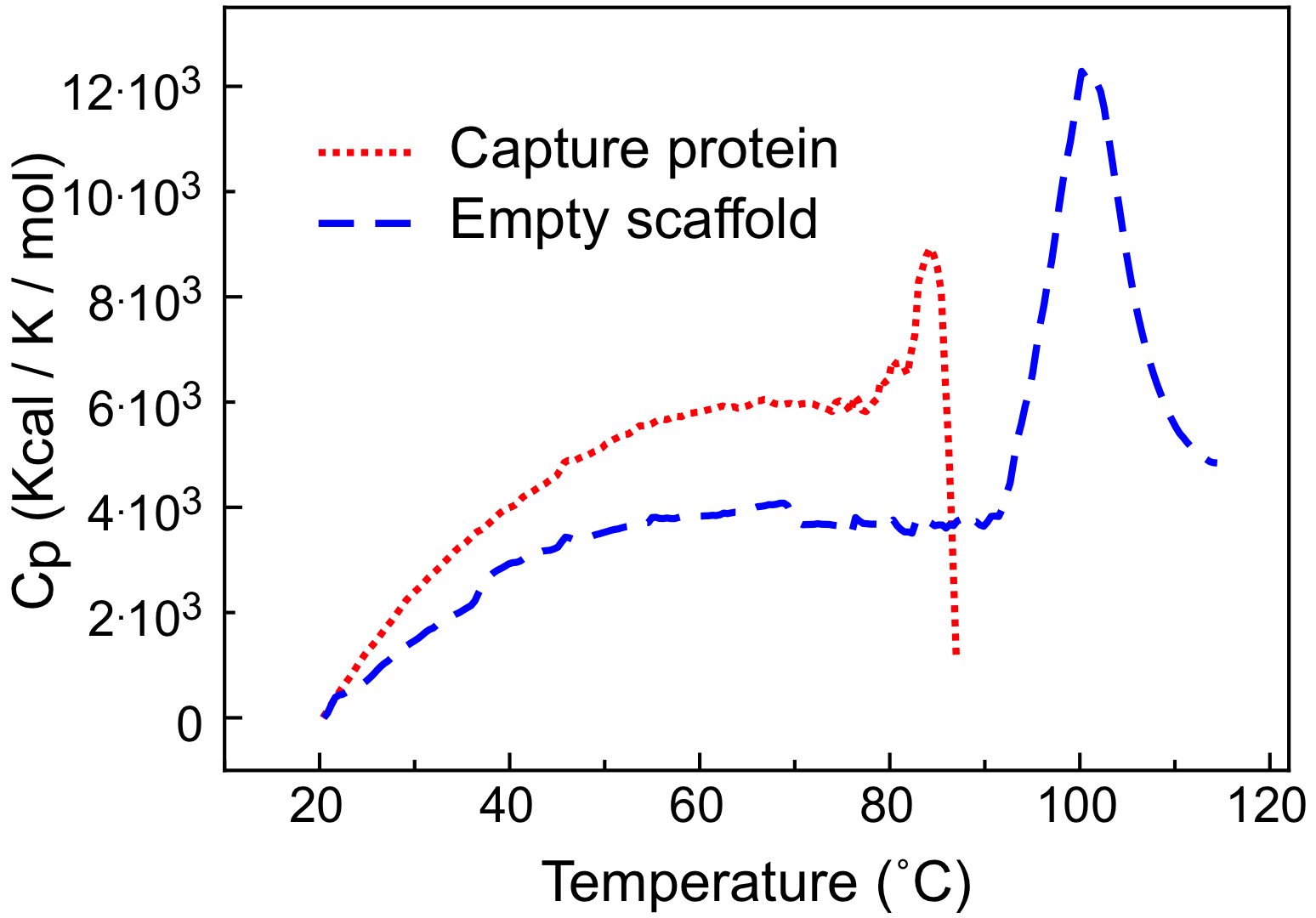


**Fig. S3** Heat capacity as a function of temperature showing a melting temperature *T_m_* of 82 ˚C for the non-antibody capture protein, compared to 101 ˚C for the empty scaffold (i.e. the scaffold protein alone without binding loops inserted).


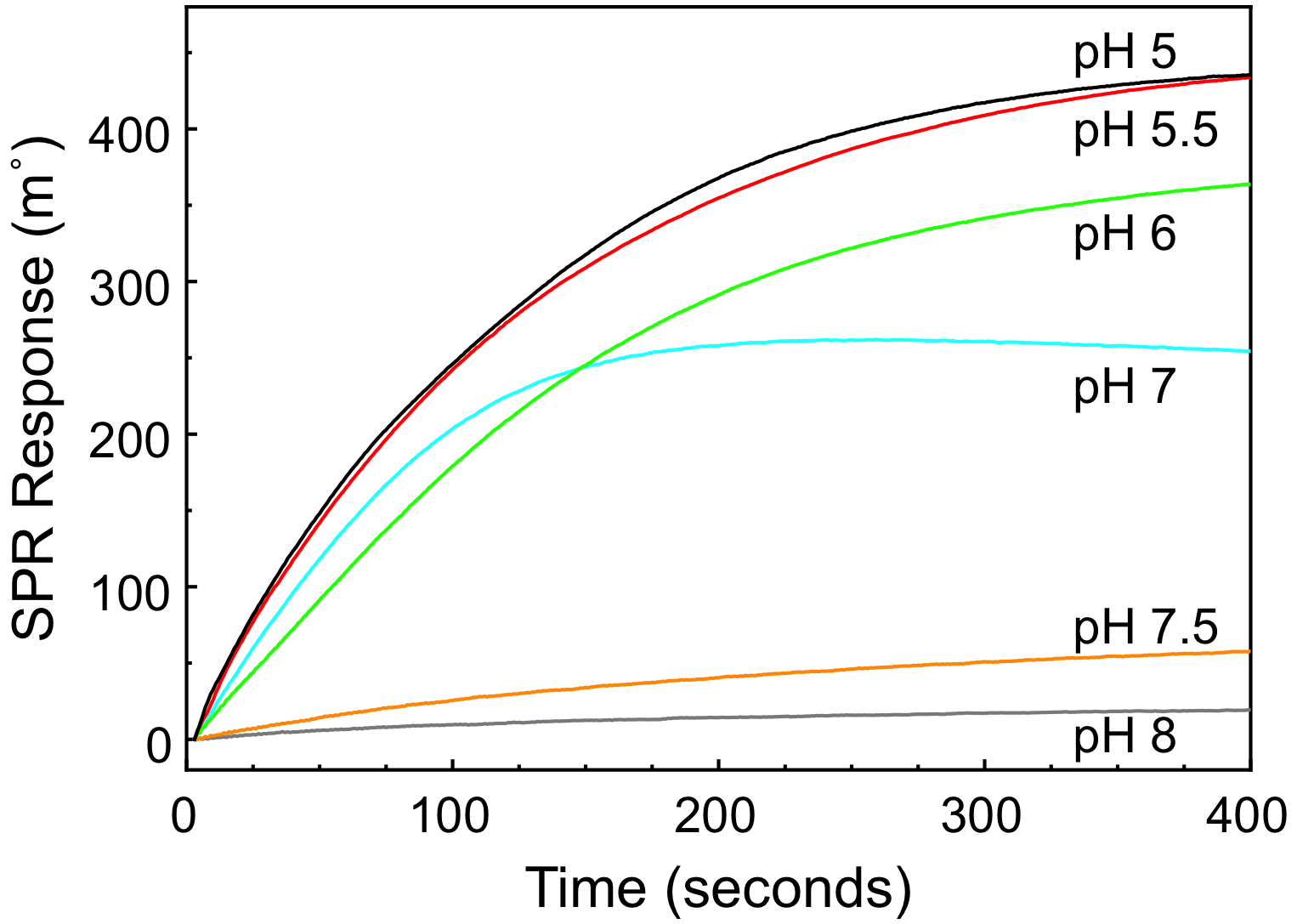


**Fig. S4** SPR sensograms recorded to determine optimal coupling conditions for binding proteins as a function of pH onto the monoalkane-thiol-PEG acid monolayer surface on a gold electrode. 10 mM phosphate buffer was used for pH 8–6, and 10 mM acetate buffer was used for pH 5.5–4.5.


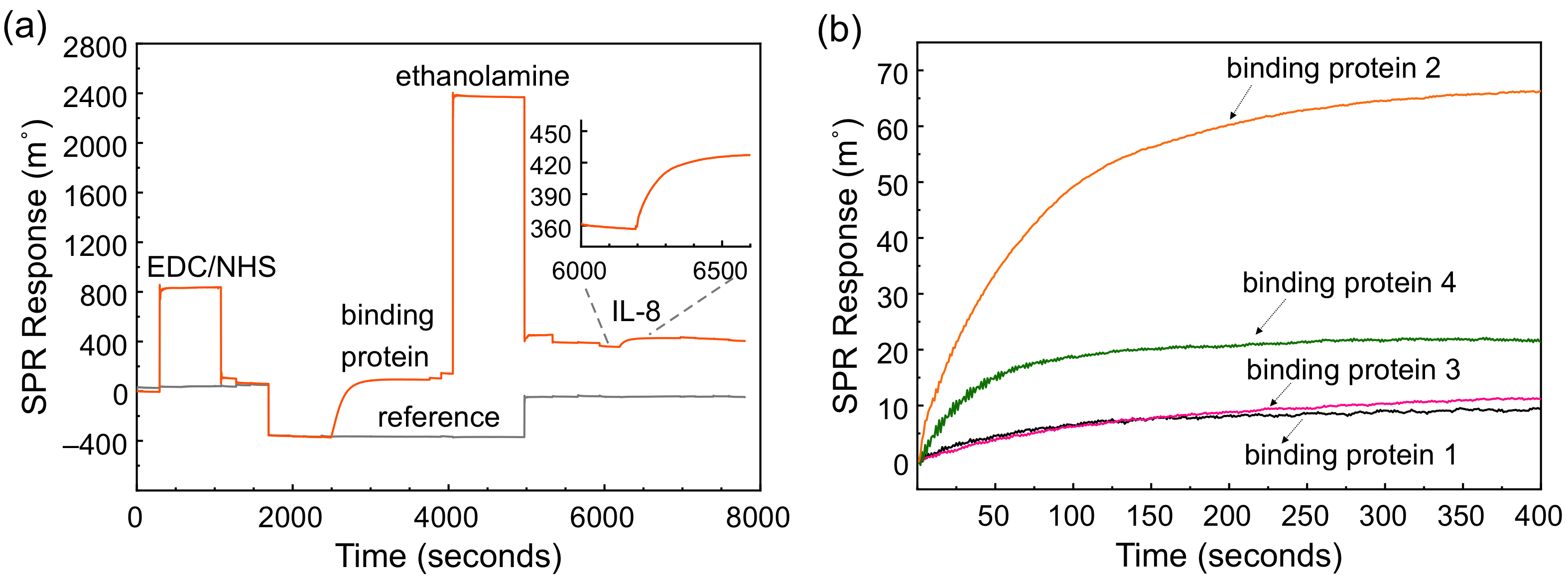


**Fig. S5** SPR sensograms showing (a) the immobilisation of the non-antibody binding proteins on the SAM functionalised gold electrode, and the binding of human IL-8 proteins at 1 µg/ml in 100 mM phosphate buffer pH 7, and (b) the binding of IL-8 proteins at 1 μg/ml concentration in 100 mM phosphate buffer pH 7 to sensor surfaces functionalised with four different non-antibody binding proteins selected via phage display. The binding protein showing the highest SPR response (binding protein 2) was chosen as the capture molecule for the subsequent biosensor work.

**
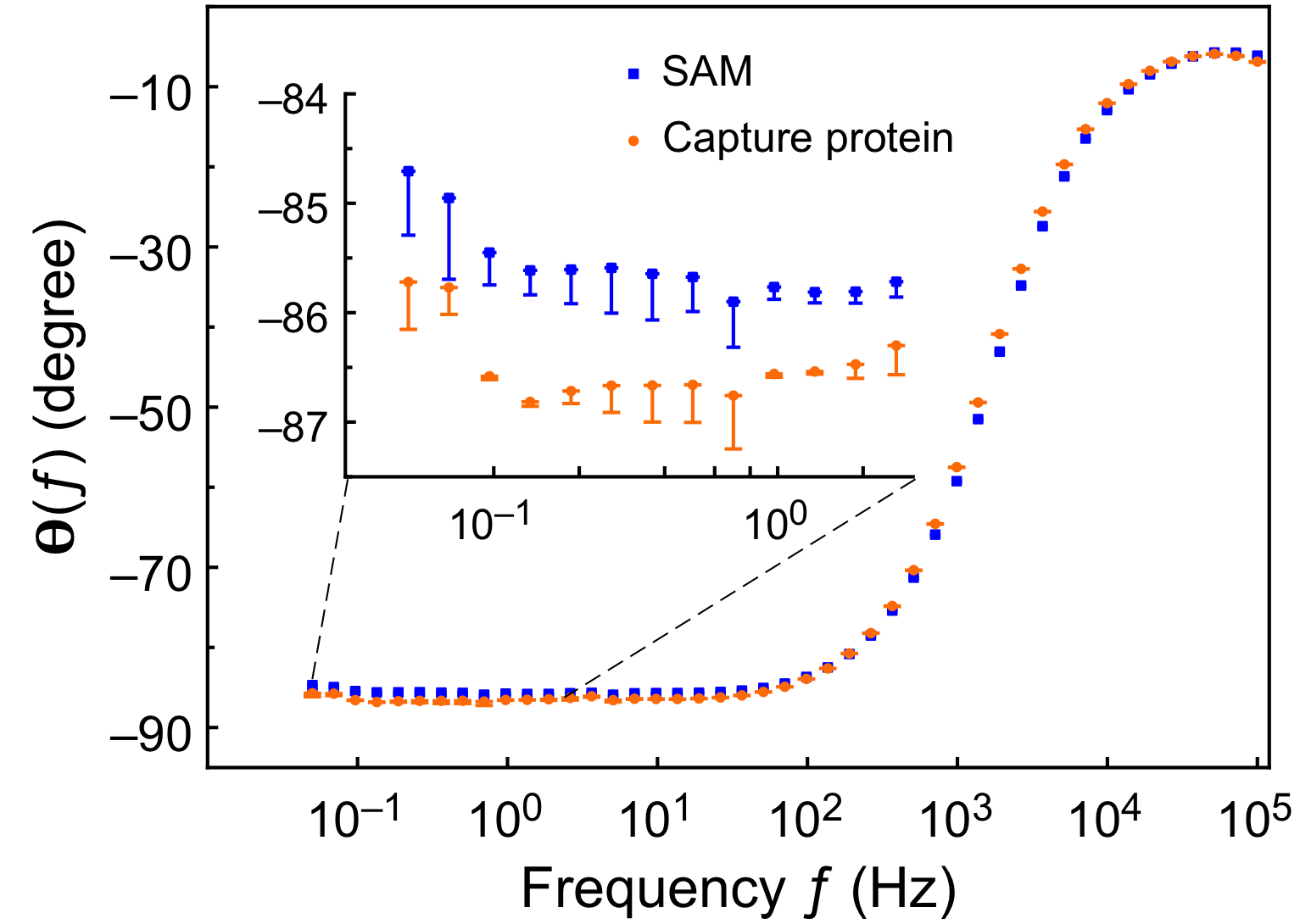
**

**Fig S6:** EIS Bode plot showing θ(ƒ) upon immobilisation of the non-antibody capture proteins on the SAM at a dc offset of 0 mV vs Ag/AgCl. The data shown represent the average of five EIS scans.

**
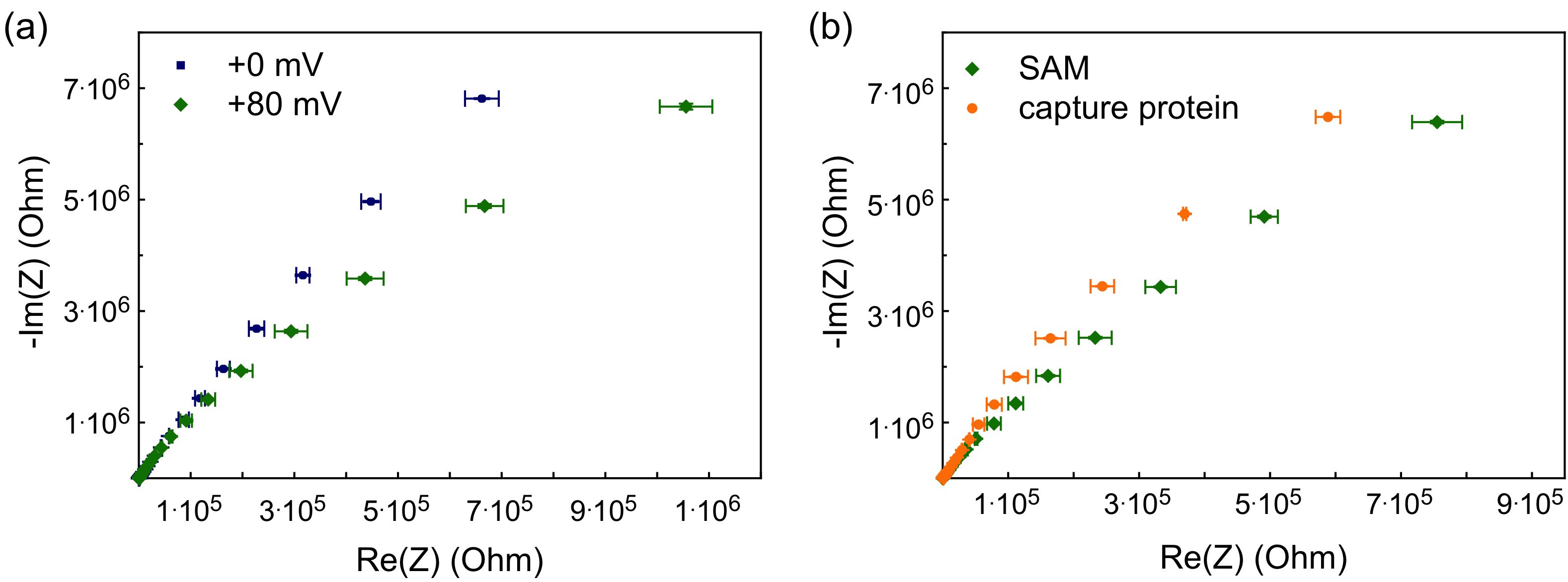
**

**Fig S7:** Nyquist plots showing the imaginary versus the real impedance of the sensor after (a) the formation of the monothiol-alkane-PEG acid SAM on the gold electrode at 0 mV and +80 mV dc potential, and (b) the immobilisation of the non-antibody capture molecules on the SAM in comparison to the SAM only at a dc offset potential of +80 mV vs Ag/AgCl. The EIS measurements were conducted in 100 mM phosphate buffer at pH 7 and the data shown represent the average of five EIS scans.

**
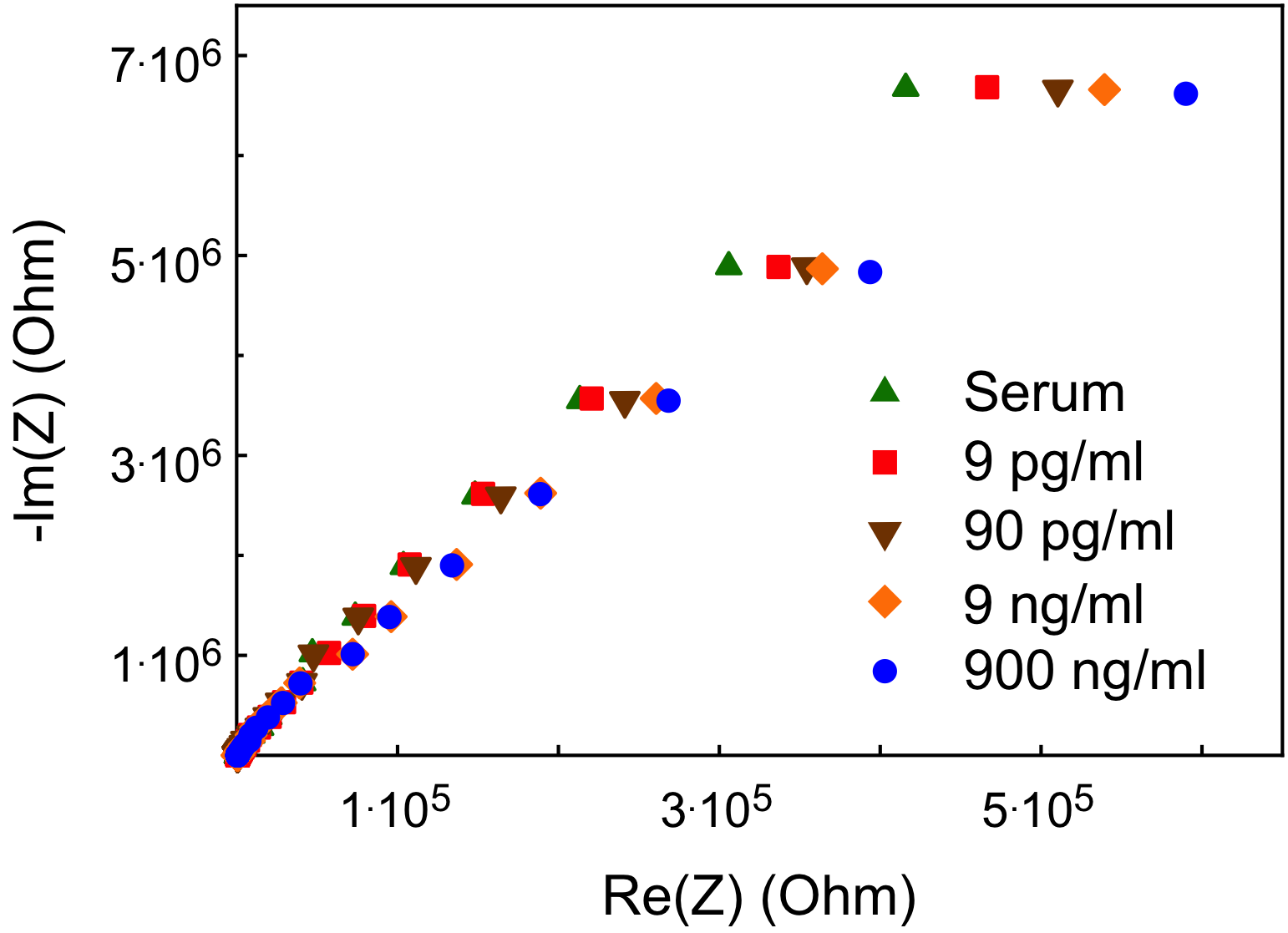
**

**Fig S8:** Nyquist plot showing the change in response of the sensor from the baseline response vs IL-8 concentration between 9 fg/ml and 900 ng/ml.


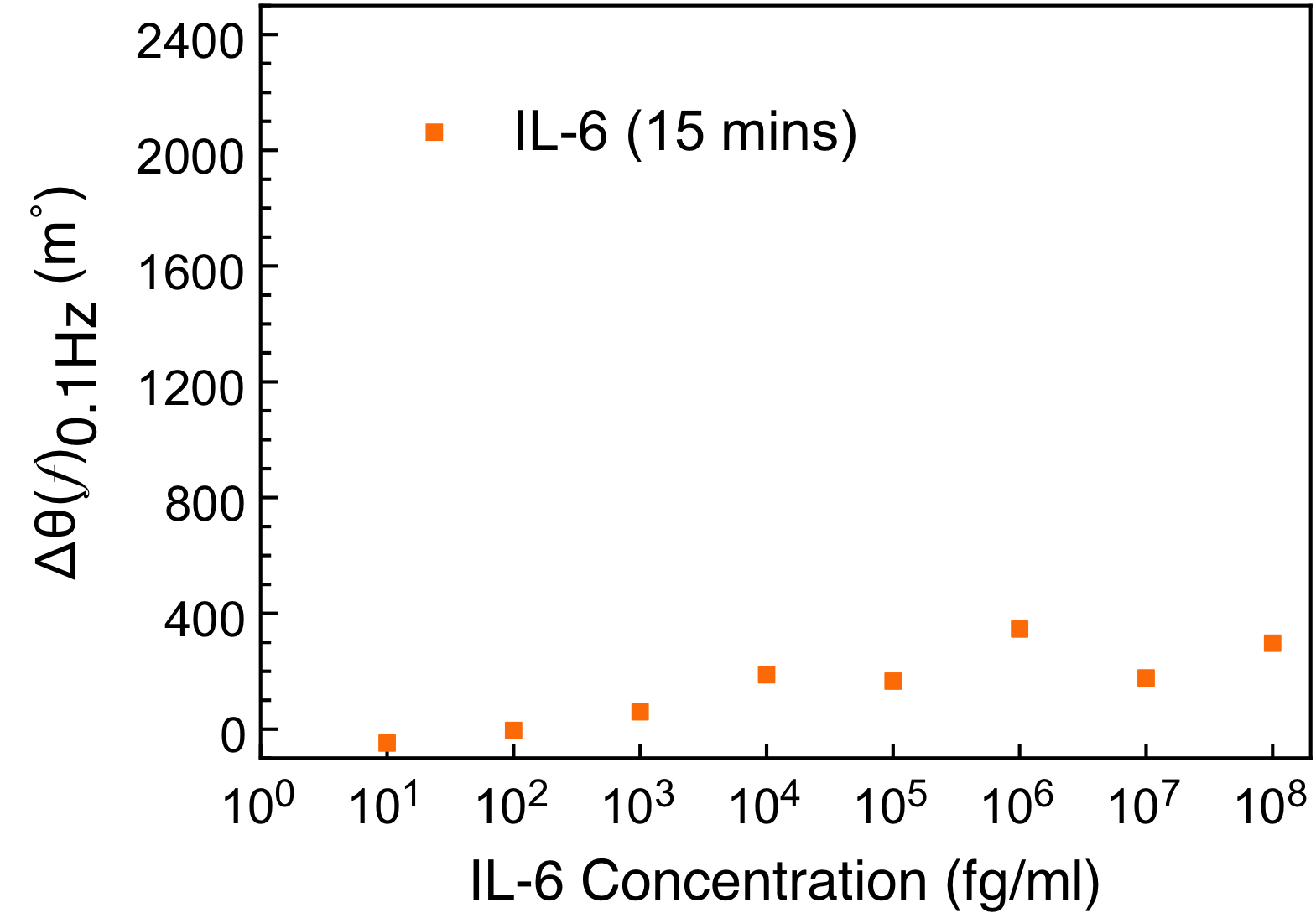


**Fig S9:** EIS sensogram showing the change in phase from the baseline at 0.1 Hz, ∆θ(ƒ)_0.1Hz_, of the sensor response when exposed to human IL-6 in 100 mM phosphate buffer pH 7 at concentrations between 10 fg/ml and 100 ng/ml. All EIS scans were performed at a dc offset of +100 mV vs Ag/AgCl.
